# Tea and Its Active Ingredients in Preventing and Alleviating Depression: A Comprehensive Review

**DOI:** 10.3390/foods14122054

**Published:** 2025-06-11

**Authors:** Shuangling Xiao, Yi Li, Haiyan Jiang, Sitong Hou, Yaoyao Wang, Di Wang, Jie Teng

**Affiliations:** 1 School of Land Resources and Environment, Jiangxi Agricultural University, Nanchang 330045, China; xiaosl@jxau.edu.cn (S.X.);; 2 College of Agriculture, Jiangxi Agricultural University, Nanchang 330045, China

**Keywords:** *Camellia sinensis*, depression, functional component, HPA axis, GABA, EGCG, tea polyphenols, neuroscience

## Abstract

**:** Depression, commonly known as unipolar affective disorder, is one of the most prevalent mental illnesses in contemporary society, affecting individuals to varying degrees. Tea is one of the three major non-alcoholic beverages globally; it has a rich history of consumption and is associated with numerous health and nutritional benefits. This review systematically summarizes the antidepressant effects of various bioactive compounds found in tea, drawing upon research findings in the field of tea’s functional health. It elucidates the impact of tea’s bioactive components on the hypothalamic–pituitary–adrenal (HPA) axis, the nervous system, the immune system, intestinal microflora, and the monoaminergic system, among other physiological sites, to achieve antidepressant effects. These effects primarily involve enhancing neural signaling pathways, regulating neural signaling molecule levels, and reducing neuroinflammation. Tea may normalize the body’s nervous system by bolstering immune function, alleviating or eliminating cellular inflammation to maintain healthy homeostasis, or improving intestinal flora and mitigating stress to prevent or treat depressive disorders. Additionally, the potential social support derived from tea-drinking activities, such as cultural rituals and interpersonal communication, may contribute to its antidepressant effects. This review discusses and analyzes the current research status regarding the antidepressant effects of tea and highlights that tea and its active ingredients can be utilized to prevent and alleviate depression.

## 1. Introduction

The clinical manifestations of depression include persistent depressive moods, accompanied by cognitive, behavioral, or autonomic nervous system abnormalities [1]. These symptoms significantly impair the personal health of patients and exert considerable pressure on society [2]. In particular, the global outbreak of COVID-19 has resulted in increased isolation and medical gaps, further exacerbating the prevalence and stress associated with major depressive disorder [3]. According to statistics, more than 350 million people worldwide suffer from depressive symptoms of varying degrees [4]. Numerous interventions are available for the treatment of depression, including medication, psychotherapy [5], and exercise therapy [6]. Research indicates no significant difference in efficacy between medication and psychotherapy [7]. However, psychotherapy often imposes a substantial financial burden on patients [8] and is associated with certain adverse effects [9]. Additionally, both psychotherapy and pharmacological treatments may only partially alleviate symptoms. Consequently, this research aims to explore alternative methods for the prevention and treatment of depression [10]. Recent scientific investigations have underscored the positive impact of healthy lifestyles, particularly dietary choices, on the prevention and management of depression [11]. Evidence suggests that the quality and type of diet are correlated with depression [12,13]. For instance, long-term alcohol consumption and cigarette smoking are linked to an increased risk of depression [14]. Furthermore, studies indicate that vegans may face a higher risk of developing depression compared to omnivores [15]. Additionally, the consumption of soft drinks has been associated with an increased risk of depression [16]. In contrast, tea has significant research potential as a beverage for its role in the prevention or alleviation of depression.

Tea, one of the three major non-alcoholic beverages globally, is derived from the leaves of *Camellia sinensis* and is widely appreciated by consumers around the world [17]. It contains a diverse array of biologically active compounds, including tea polyphenols, theanine, caffeine, and catechins, which contribute to its various health benefits [18]. Studies have confirmed that tea consumption has both stimulating and calming effects on the human brain and nervous system, enhancing cognitive function and regulating mood [19,20]. Tea could decrease the risk of depressive symptoms by reducing oxidative stress and inflammatory processes, regulating intracellular signaling pathways, modulating the hypothalamic–pituitary–adrenal (HPA) axis, and influencing the levels of monoamine neurotransmitters and metal chelation [21,22]. Furthermore, tea consumption is associated with beneficial effects on mental health [23]. This article reviews the potential mechanisms of tea and its biologically active ingredients in the prevention and treatment of depressive disorders, drawing on observational and clinical research.

## 2. The Etiological Mechanism of Depression and the Antidepressant Effects of Tea Components

### 2.1. Etiological Mechanism of Depression

**Figure 1:**
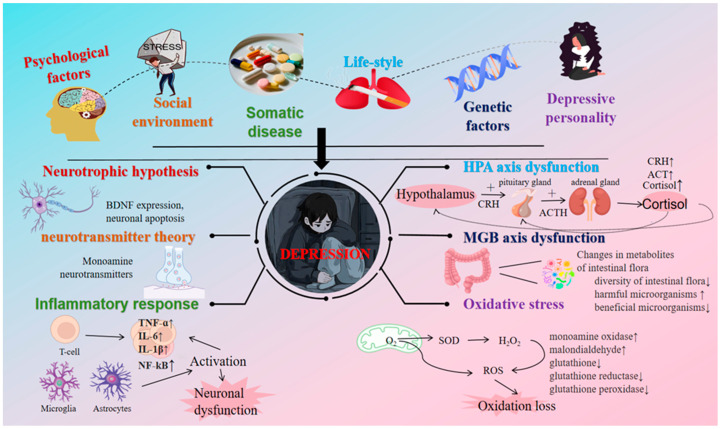
The main factors influencing depression and their pathological mechanisms.

The advancement of neuroscience and technology, coupled with the maturation of theories in related biological and physiological disciplines, has enabled researchers to gain a profound understanding of the organizational structure of the human brain and its functional principles [24]. This progress has also facilitated extensive research into the pathogenesis of depression. However, the mechanisms underlying depression are complex, and its precise causes remain to be accurately identified [25]. As of today, researchers have explored the potential pathogenesis of depression from multiple physiological perspectives, including the monoamine neurotransmitter and receptor hypothesis [26], the HPA axis dysfunction hypothesis [27], the neural plasticity theory and neurotrophic factor hypothesis [28], the cellular molecular mechanism hypothesis [29], the neuroimmunity and cytokines hypothesis [30], the excitatory amino acid hypothesis [31,32], the intestinal flora disorder hypothesis [33], as well as the influence of social environment and genetic factors [34,35]. The prevailing hypothesis concerning monoamine neurotransmitters and their receptors represents the current mainstream perspective on the etiology of depression. However, analyzing the etiology and mechanisms of depression solely from a reductionist viewpoint is overly simplistic, and the external validity of the psychopathology observed in clinical treatment remains limited. A comprehensive theory of depression’s etiology highlights the interconnections among neurobiological systems involved in its pathology [36], including the microbiota–gut–brain axis (MGBA) [37,38]. This perspective suggests that within the extensive network of physiological, psychological, and social environments, multiple pathogenic factors interact, potentially triggering depression through abnormal functions in specific aspects of the patient’s body [36] (Figure 1). Nonetheless, the roles and mechanisms of each pathogenic factor in the pathogenesis of depression require further clarification.

Today, a variety of medical drugs have been developed for the treatment of depression. Typical antidepressants include monoamine oxidase inhibitors (MAOIs), tricyclic antidepressants (TCAs), and newly developed selective serotonin reuptake inhibitors (SSRIs), as well as serotonin/norepinephrine reuptake inhibitors (SNRIs), norepinephrine/dopamine reuptake inhibitors (NDRIs), and serotonin antagonist-reuptake inhibitors (SARIs). However, these antidepressants may exhibit limitations, such as restricted efficacy, side effects, and withdrawal symptoms. These shortcomings ultimately arise from the abnormal interactions of various monoamine neurotransmitters with the drugs. In contrast, tea contains numerous bioactive compounds that may exert antidepressant effects through mechanisms that extend beyond the traditional monoamine pathway. Consequently, studying the relationship between the effects of individual compounds in tea and their ability to treat depression or alleviate depressive symptoms poses significant challenges. This is primarily due to the fact that no single compound in tea can precisely and effectively inhibit monoamine reuptake or antagonize specific monoamine receptors, similar to the various established antidepressants [25]. Various bioactive compounds in tea interact synergistically across multiple nervous systems, contributing to the well-documented antidepressant effects of tea. Additionally, many researchers have developed novel glutamatergic drugs, γ-aminobutyric acid (GABA) medications, and other pharmacological agents to further enhance the network of treatments available for depression [39].

### 2.2. Research Approaches

The primary research methods currently employed involve applying the bioactive ingredients found in tea to animal models to investigate the pathophysiology associated with depression and to establish the relationship between tea compounds and depression. However, it is crucial to acknowledge the limitations inherent in using animal models, as the multifactorial nature of human depression, such as genetic variability, psychosocial stress, and verbal support, renders it challenging to replicate in animal studies [40]. Therefore, it is essential to consider the external validity of tea compounds. Although human studies are still in their early stages, primarily consisting of epidemiological observational studies, there are also some clinical intervention studies. Importantly, these studies adhere to the ethical and moral principles of scientific research as well as relevant laws and regulations.

#### 2.2.1. Observational Studies

Observational studies and efficacy reports in the field of nutritional psychiatry provide substantial evidence supporting the role of healthy eating patterns in managing depressive episodes and symptoms [41]. In a related investigation of the relationship between tea consumption and depression, researchers identified an inverse relationship between depression and the intake of green tea. In the Chronic Inflammation and Health Promotion Cohort Study (TCLSIH Cohort study) conducted in China, high consumption of green tea was closely associated with reduced depressive symptoms. After adjusting for potential confounding factors, it was determined that frequent consumption of green tea (≥1 cup/day) was inversely proportional to the incidence of depressive symptoms. This study offers a compelling rationale for increasing green tea consumption as a potential preventive measure against depressive symptoms in adults in China [42]. A Japanese research team conducted a cross-sectional study to analyze the association between green tea consumption and depressive symptoms. The findings indicated that green tea consumption was inversely related to the incidence of depressive symptoms. Participants who consumed ≥4 cups of green tea daily exhibited a significantly reduced prevalence of depressive symptoms by 51% (*p* < 0.01) compared to those who consumed ≤1 cup per day, even after adjusting for potential confounding variables [43]. Similarly, a longitudinal study in Singapore investigated the relationship between tea consumption and depression. The results revealed that individuals who consumed ≥3 cups of tea were significantly less likely to experience a deterioration of GDS (Geriatric Depression Scale) symptoms (risk ratio (RR) = 0.32, 95% confidence interval (CI) = 0.12~0.84), suggesting that tea consumption may help prevent the worsening of depressive symptoms and reduce the likelihood of developing threshold depression [44]. A study conducted in South Korea utilized nationally representative large-scale data to examine the association between green tea intake and self-reported lifelong depression among 9576 adults. The findings indicated that individuals who regularly consumed green tea (≥3 cups per week) experienced a 21% reduction in the prevalence of depression compared to non-tea drinkers (RR = 0.79, 95% CI = 0.63~0.99) [45]. However, these surveys were regionally focused and conducted by research teams in Asia, which introduces certain geographical limitations. Given that Asia is characterized by a prevalent tea-drinking culture, where tea consumption is not only a dietary habit but also a symbol of social etiquette, various cultural factors may skew the accuracy of the survey data. Conversely, a survey conducted in the United States revealed no association between the consumption of iced tea or hot tea and the risk of depression [46]. Additionally, research from Finland also found no correlation between tea intake and depression. After adjusting for age and follow-up duration, the risk of depression among tea drinkers (2150 cases) compared to non-tea drinkers (82 cases) appeared to decrease. This limitation primarily arises from the restricted methods of daily diet control among the subject group, necessitating further exploration of the experimental conclusions [47]. Nonetheless, a significant limitation of observational studies is their inability to establish causal relationships; they can at best demonstrate strong correlations. Thus, only through multi-region collaborations and randomized controlled clinical trials can we elucidate the physiological and biochemical mechanisms by which the functional components of tea exert antidepressant effects. This approach will ultimately lead to conclusions that support the significant causal impact of tea and its constituents on depression.

#### 2.2.2. Intervention Studies

Conducting human clinical experimental studies often presents challenges due to the constraints imposed by social ethics, laws, and regulations. Consequently, designing a randomized placebo-controlled trial to evaluate the effects of tea and its components on depression has proven difficult. Nevertheless, some research teams have achieved robust experimental results. For instance, a clinical study conducted in Iran randomly assigned 72 subjects at the Rasht Elderly Center using simple random sampling while controlling for various demographic characteristics. The experimental intervention lasted for 5 weeks, during which participants consumed 3 g green tea twice daily, after breakfast and lunch. The findings indicated that drinking green tea significantly reduced the severity of depression among the elderly (*p* < 0.01), and the number of cases of severe depression decreased following the intervention [48]. However, the absence of a placebo treatment in this study limited its reliability and validity. Similarly, an African study employed a pre-and-post design without a placebo group. In this study, researchers randomly selected 491 white-collar employees who consumed at least 100 mL of black tea daily. The results indicated that when participants ingested an average of 629.5 ± 418.8 mg of caffeine from black tea per day, it exhibited a protective effect against depression. Furthermore, after adjusting for potential confounding factors, subjects who consumed one to four cups of black tea per day demonstrated a lower prevalence of depression [49]. A recent research experiment conducted in Japan randomly recruited 30 healthy subjects without significant mental illness. The experimental procedure involved the random and blind allocation of either *L*-theanine (200 mg/day) or placebo tablets over a four-week period. The results indicated that the subjects’ scores on the Self-Rating Depression Scale decreased by 0.019 after the administration of L-theanine, suggesting that *L*-theanine may have a positive impact on the mental health of the general population [50]. However, there are limited data regarding the application of teas and their constituents in clinical trials. Due to the scarcity of advanced clinical studies, definitive conclusions regarding the preventive and therapeutic effects of tea on depression cannot be drawn. Therefore, it is essential to establish a placebo-controlled trial to validate the antidepressant effects of compounds found in tea.

### 2.3. Mechanism of Tea’s Action on Depression

Numerous biologically active ingredients have been identified in tea and its processed products, including polyphenols and their oxidation products, theanine, purine alkaloids, aromatic compounds, carbohydrates, and saponins. The concentrations of these compounds vary among different types of tea [51]. These diverse chemical constituents contribute to tea’s antioxidant activity, anti-inflammatory effects, regulation of the immune system, neuroprotective properties, and other health benefits [52]. Within the context of depression pathology, various bioactive components of tea interact at different biophysical network nodes, and certain tea components exert bioactive effects across multiple physiological systems. This section provides a concise overview of the current research regarding the antidepressant properties of tea, focusing on its constituent ingredients (Table 1).

#### 2.3.1. L-Theanine

*L*-theanine is an important functional component found in tea. Upon consumption, tea induces various biochemical mechanisms in the body that help prevent or mitigate psychological stress. Furthermore, research indicates that *L*-theanine may be associated with the treatment of depression.

##### L-Theanine Regulates the HPA Axis

*L*-theanine has been shown to mitigate abnormalities in HPA axis activity, thereby achieving antidepressant efficacy. Research indicates that *L*-theanine treatment (20 mg/kg/day) significantly reduces the elevation of adrenocorticotropin (ACTH) and cortisol (CORT) in mouse models subjected to inflammatory stress via lipopolysaccharide (LPS) administration. This effect is believed to result from *L*-theanine’s ability to normalize HPA axis function and attenuate the phosphorylation of nuclear factor kappa-light-chain-enhancer of activated B cells (NF-κB) in liver tissue while also modulating the inactivation of the NF-κB signaling pathway [53]. Additionally, other studies suggest that the mechanism underlying *L*-theanine’s impact on the HPA axis may involve its antagonistic effects on glutamate, a key component in the excitatory regulation of HPA axis activity [104]. At the post-synaptic level, glutamate is implicated in driving the acute HPA axis stress response through the activation of alpha-amino-3-hydroxy-5-methyl-4-isoxazolepropionic acid (AMPA) receptors. Conversely, at the presynaptic level, glutamate inhibits corticotropin-releasing hormone (CRH) neurons through both ionotropic (GluR5) and metabotropic (group 1 mGluR) mechanisms, thereby modulating HPA axis activity [105]. *L*-theanine is absorbed in the intestine and crosses the blood–brain barrier (BBB), where it demonstrates a strong affinity for the glutamine transporter, suggesting a significant interaction between the two. It can reduce the release of glutamate from presynaptic neurons into the synaptic cleft, thereby inhibiting neuronal hyperexcitability [54]. However, the extent to which GABA synthesized outside the brain can penetrate the BBB remains uncertain, primarily due to the difficulties associated with measuring GABA levels in the human brain. This uncertainty has led to ongoing debate among researchers [106]. Nevertheless, the increase in GABA levels in the brain significantly contributes to the validation of theanine’s anti-irritating effect and the normalization of HPA axis activity.

##### Neuroregulatory Mechanism of L-Theanine

*L*-theanine (0.2 μmol/2 μL perfusate/min, injected for 1 h) directly interacts with ionic glutamate receptors in the brain, particularly AMPA receptors, which are responsible for transmitting most excitatory signals in the central nervous system (CNS) through mediated nodes. Studies indicate that *L*-theanine preferentially acts on AMPA receptors rather than on N-methyl-D-aspartate (NMDA) receptors, resulting in an increase in glycine concentration within the striatal stroma [55]. Glycine serves as the primary inhibitory neurotransmitter in the spinal cord and brainstem, playing a crucial role in processing motor and sensory information [107]. Additionally, glycine functions as a co-agonist of the NMDA receptor, which appears to regulate dopamine (DA) release, though this relationship remains contentious [108]. The interplay between *L*-theanine, glycine, and glutamic acid may partially elucidate the mood-regulating effects of *L*-theanine [109]. One study demonstrated that administering 2 mg/kg of *L*-theanine ameliorated depression-like behavior in a rat model of chronic unpredictable mild stress (CUMS). In the prefrontal cortex (PFC), nucleus accumbens (NAc), amygdala (AMY), and hippocampus (HIP) of these rats, *L*-theanine significantly elevated levels of 5-hydroxytryptamine (5-HT), norepinephrine (NE), and DA [56]. It is well established that increases in these neurotransmitters can enhance neural excitability. Furthermore, researchers observed that 5-HT and DA levels were markedly elevated in the ST, while in the HIP, only DA levels exhibited significant changes post treatment [56]. In a mouse model of Alzheimer’s disease (AD), *L*-theanine enhances monoamine transport within the HIP by activating the dopamine D1/5 receptor-PKA pathway [57]. This finding provides theoretical support for the promotion and application of *L*-theanine from the perspective of the monoaminergic hypothesis. A decreased expression of neuron PAS domain protein 4 (NPAS4) in the HIP has been associated with anxiety and depression-like behaviors [110]. Research indicates that NPAS4 facilitates the development of inhibitory GABA synapses in excitatory pyramidal cells of the HIP and functions as a transcription enhancer that regulates neuronal excitability and inhibition. Studies demonstrate that rats exhibiting higher NPAS4 expression show greater resistance to stress [111]. In an experiment utilizing a low-stress model induced by housing stress, researchers observed that the intake of *L*-theanine significantly inhibited the expression of stimulant-bound glucocorticoid receptors and DNA methyltransferase 3A (DNMT3a), which subsequently inhibited the transcription of NPAS4. By inhibiting holo-GR and DNMT3a, *L*-theanine elevates NPAS4 expression, potentially adjusting the excitation/inhibition balance and thereby reducing the stress response [58].

In addition, *L*-theanine has been shown to enhance both sleep duration and quality. Given the evidence that sleep disorders may precede the onset of depression [112], *L*-theanine could provide therapeutic benefits by improving patients’ sleep conditions. Research indicates that the administration of 200 mg of *L*-theanine enhances sleep quality by reducing intermittent arousal, thereby increasing overall sleep percentage and efficiency. The no observable adverse effect level for the oral administration of L-theanine was determined to be above 2000 mg/kg/day. Notably, *L*-theanine does not induce sleep, suggesting that its mechanism for improving sleep quality differs from that of sedatives and sleep inducers, instead promoting sleep quality in a more subtle manner [59]. Furthermore, a GABA/*L*-theanine mixture (100/20 mg/kg) demonstrated a decrease in sleep latency (20.7% and 14.9%) and an increase in sleep duration (87.3% and 26.8%) compared to GABA or *L*-theanine alone [113,114]. Additionally, studies have demonstrated that the simultaneous use of *L*-theanine or arginine with (-)-epigallocatechin-3-gallate (EGCG) or caffeine may result in an antagonistic effect, potentially diminishing the anti-stress properties of *L*-theanine [115]. Arginine, one of the most abundant free amino acids found in green tea, has been shown in various studies to possess physiological effects that alleviate stress and promote relaxation [116]. The results of this experiment indicate that the simultaneous consumption of specific proportions of caffeine or EGCG alongside *L*-theanine or arginine may lead to inhibition, thereby diminishing the efficacy of the latter. However, the anti-stress effects of *L*-theanine remain significant. Additional research has demonstrated that *L*-theanine can mitigate D-galactose-induced brain damage in rats by inhibiting the formation of advanced glycation end products (AGEs) and modulating the signaling pathways of Sirtuin1 and brain-derived neurotrophic factor (BDNF) [60]. BDNF is a key neurotrophic factor that provides essential support for the growth, development, and survival of neurons. It is involved in various intracellular signaling processes and can stimulate the release of neuropeptides and neurotransmitters at inflammatory sites in neurological diseases, influencing the immune response of the nervous system. Notably, reduced levels of BDNF are among the most prevalent and effective biomarkers for depression [117], while the upregulation of BDNF mRNA expression in the HIP and cortex resembles the antidepressant response observed with traditional antidepressants such as selective serotonin reuptake inhibitors (SSRIs) [118]. Consequently, this study offers new practical insights into the potential of *L*-theanine to exert antidepressant effects through the regulation of the BDNF signaling pathway.

##### L-Theanine and the Immune System

*L*-theanine has been shown to exert certain effects in the prevention and treatment of depression by enhancing the body’s immune system and mitigating cellular inflammation. Numerous studies indicate that levels of inflammatory cytokines, including interleukin (IL)-1β, IL-6, tumor necrosis factor alpha (TNF-α), and C-reactive protein (CRP), are elevated in patients suffering from depression [119]. Concurrently, a substantial body of experimental evidence supports a strong association between inflammatory cytokines and depression. These cytokines can influence the monoaminergic and glutamatergic systems, thereby contributing to the overlapping pathological mechanisms underlying depression in affected individuals [120]. From the perspective of the neuroimmune hypothesis, research has demonstrated that *L*-theanine can attenuate various forms of cellular inflammation, including respiratory inflammation [61] and acute skin inflammation [62].

The gut–brain axis is recognized as a crucial mechanism underlying brain injury, neuroinflammation, and related diseases. This axis constitutes a bidirectional communication network linking the CNS with the enteric nervous system (ENS) [121]. Its associated pathways predominantly depend on microbiota signaling to mitigate nervous system disorders. The intestine, rich in immune cells and intricate nerve networks, plays a significant role in facilitating signal transmission and functional interactions between the nervous and immune systems [122].

Research indicates that the diversity of microbial communities influences the development and migration of immune cells, leading to inflammatory processes such as neuroinflammation and amyloid deposition, as well as physiological processes involving BDNF and NMDA signal transduction, which may trigger or exacerbate neuroinflammation and neurodegenerative diseases. Notably, a study demonstrated that oral *L*-theanine at a concentration of 0.1% (*w*/*v*) administered for 30 consecutive days mitigated colitis induced by 2% (*w*/*v*) sodium gluconate (DSS). This treatment alleviated inflammation-related pathological damage in the colon and improved the histopathological features of DSS-induced colitis; it also inhibited the DSS-induced alterations in colon tissue. Furthermore, when compared to the DSS induction group, the treatment group exhibited an 85% increase in GSH levels, a 55% reduction in LPS levels, and significant decreases in interleukin (IL)-1β, IL-6, and TNF-α levels [64]. A wealth of experimental evidence supports the regulatory role of *L*-theanine on the immune system, modulating inflammatory cytokine levels within the physiological system. This provides a foundation for future research to further investigate the antidepressant effects of *L*-theanine through the lens of the neuroimmunity hypothesis.

#### 2.3.2. GABA

GABA is a unique non-protein amino acid, and its potent antidepressant effects have long been recognized. As a crucial inhibitory neurotransmitter in the CNS, GABA plays a stabilizing role during periods of stress. Under normal circumstances, the synthesis and secretion of cortisol (CORT) in the human body trigger an inhibitory mechanism within the corticotropin-releasing factor (CRF) signaling pathway, which reduces the activity of the HPA axis, thereby facilitating stress regulation. The primary inhibitory mechanism of GABA involves GABAergic signaling activated within negative feedback microcircuits [123]. When CORT binds to glucocorticoid receptors and mineralocorticoid receptors (MRs) in vivo, GABA inhibits glutamate release and diminishes the firing of CRF neurons [65]. As glutamatergic activity decreases, the expression of GABA receptors on CRF neurons increases, enabling corticotropin-releasing hormone receptor 1 (PVNCRFR1) neurons in the paraventricular nucleus of the hypothalamus to form recurrent GABAergic synaptic connections with active CRF neurons [66]. Consequently, transsynaptic GABAergic signaling suppresses the excitability of CRF neurons, reduces HPA axis activity, prevents dysregulation of this axis, and maintains homeostasis [66,124].

**Figure 2:**
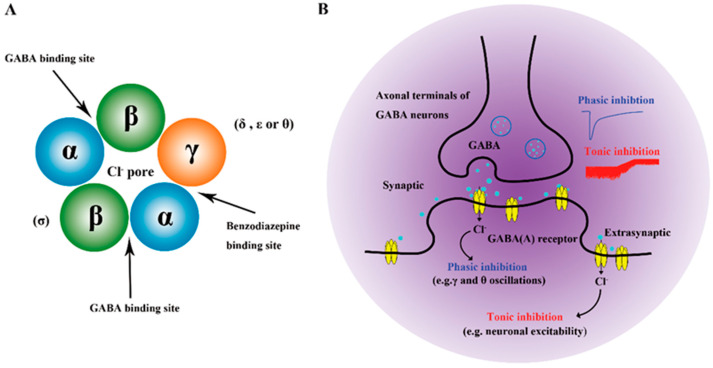
(**A**) The structure and GABA/benzodiazepine binding sites of GABAA receptors, (**B**) phasic versus tonic inhibition of the postsynaptic membrane mediated by synaptic and extrasynaptic GABAA receptors.

Currently, GABA is recognized for its action on three subtype receptors: GABAA, GABAB, and GABAC. The GABAA and GABAC receptor complex is a pentameric structure composed of multiple protein subunits in a stoichiometric ratio of 2:2:1, which forms an ion channel arranged around a central chloride channel. This receptor complex contains not only the GABA binding site located between the α and β subunits but also the binding site for substances that modulate the effect of GABA. Upon activation of the GABAA/C receptor, Cl^−^ influx is enhanced, resulting in membrane hyperpolarization that inhibits the transmission of electrical signals in neurons, thereby reducing neuronal activity. In contrast, the GABAB receptor is a metabolic receptor, functioning as a heterodimer composed of GABAB1 and GABAB2 subunits. These two receptor proteins are coupled through their C-terminal domains and are embedded in the plasma membrane, originating from the endoplasmic reticulum as a heterodimer. The GABAB1 subunit provides the GABA-binding domain in its outer chain, while GABAB2 facilitates G-protein coupling, which links them to Ca^2+^ or K^+^ channels. When activated, these channels enhance K^+^ conductance within the membrane or suppress Ca^2+^ current, resulting in decreased electrical conductivity and subsequent hyperpolarization of neurons. Early researchers have highlighted the potential of GABAB receptors as targets for antidepressant therapies [125]. In both mechanisms, GABA modulates neuronal signal transmission to regulate nerve cell activity [67], contributing to its antidepressant effects, as illustrated in Figure 2 [126]. Currently, GABA-based antidepressant drugs are in the clinical trial phase, where researchers are evaluating and optimizing their safety, tolerability, long-term effectiveness, and overall efficacy. It is anticipated that these drugs could emerge as novel monoaminergic antidepressants in the future [127].

In the preceding discussion, we examined the mechanisms by which GABA exerts its depressive regulatory effects on the nervous system. Additionally, the combined regulatory influence of GABA and intestinal microbial flora on the nervous system warrants further attention. Research indicates that *Corynebacterium* spp., *Streptococcus* spp., and *Escherichia coli* are capable of synthesizing 5-HT during culture [128]. Furthermore, *Lactobacillus* [129], *Bifidobacterium* [130], and *Bacteroides* spp. [68] can secrete GABA, suggesting that the intestinal microbiota may facilitate neuroregulatory effects through the production of both GABA and 5-HT. Notably, studies have demonstrated that long-term administration of *Lactobacillus rhamnosus* JB-1 (*L. rhamnosus*) enhances GABA receptor expression in the AMY and HIP, which in turn reduces stress-induced CORT levels, stress, and depressive behaviors [68]. Another investigation revealed that the relative abundance of Bacteroides, a primary GABA producer in the human gut, is negatively correlated with the functional connectivity between the left dorsolateral prefrontal cortex and the Default Mode Network. This network, which encompasses the left anteromedial frontal cortex, is closely associated with the limbic system and plays a crucial role in emotion regulation [68]. Overall, GABA serves as the principal inhibitory neurotransmitter in the brain, and the GABAergic pathway contributes significantly to the antidepressant effects associated with the regulation of the nervous system [126,131]. Simultaneously, *L*-theanine facilitates the decarboxylation of glutamate to GABA, enhancing its efficacy [54]. Research indicates that the administration of GABA-rich green tea extract significantly reduces the duration of immobility in both the forced swimming test and the tail suspension test. Additionally, a positive correlation was observed between GABA levels in mouse brain tissue and both GABA intake and behavioral outcomes. These findings suggest that the antidepressant effect of GABA-rich green tea may be linked to an increase in GABA concentration within mouse brain tissue [132].

#### 2.3.3. Tea Polyphenols

The biologically active ingredients in tea primarily consist of four major categories of substances: a series of polyphenol compounds, including flavanols (catechins), flavonoids and flavonoid glycosides, anthocyanins, phenolic acids, and depsides. Of the total tea polyphenols, catechins account for approximately 70%, which include (-)-epicatechin (EC), (-)-epigallocatechin (EGC), (-)-epicatechin-3-gallate (ECG), and EGCG. These catechin compounds can be converted into oxidation products known as tea pigments in fermented tea, which include theaflavins (TFs), thearubigins (TRs), and theabrownins (TBs) [133]. These pigments contribute to the taste and color of various types of tea. Additionally, we explore the antidepressant effects of tea pigments. The overall mechanistic effect of tea polyphenols on depressive disorders primarily relies on ECGC, EGC, ECG, EC, flavonoids, and related compounds.

##### Flavonoids

Although flavonoids constitute only 3% to 4% of the dry matter in tea, some studies suggest that their antidepressant effects may have been underestimated [134]. It is now evident that the potential antidepressant properties of flavonoids include the quenching of free radicals, chelation of metal ions, stimulation of antioxidant enzymes in the body [135], and modulation of the type and structure of intestinal microorganisms [136]. In a study involving patients with Parkinson’s disease (PD), it was observed that flavonoids primarily function to protect neurons from oxidative stress, inhibit neuroinflammation, and interact with critical intracellular signaling pathways in neurons. These pathways encompass protein kinase and lipid kinase signaling, which significantly influence neuronal function by altering the phosphorylation state of target molecules and regulating gene expression [69]. Thus, continued consumption of tea rich in flavonoids may enhance the clinical management of depressive disorders or help prevent the onset of depressive symptoms.

##### EGCG

(1) EGCG Regulating the HPA Axis

The study hypothesizes that the anti-stress mechanism of EGCG involves the normalization of the HPA axis through the restoration of plasma CORT levels. An intraperitoneal injection of EGCG into a stressed animal model over a two-week period significantly reduced the elevation of plasma CORT levels induced by sensory processing sensitivity (SPS) stimulation. Furthermore, a 25 mg/kg EGCG injection for 14 consecutive days also diminished the increases in plasma CRH and ACTH levels resulting from SPS stimulation (*p* < 0.05) [70], suggesting that EGCG can inhibit stress hormone levels within the HPA axis, thereby facilitating its regulation. Additionally, the study demonstrated that EGCG can restore certain plasma CORT levels to normal in a mouse model of HPA dysfunction induced by four weeks of restraint stress, likely by activating the extracellular signal-regulated kinase (ERK1/2) signaling pathway. ERK1/2, a member of the mitogen-activated protein kinase family closely associated with depression, plays a crucial role in regulating neurogenesis, proliferation, and differentiation while also significantly influencing synaptic plasticity, learning, and memory. Numerous studies have indicated that mixtures of EGCG and green tea polyphenols (GTPs) can inhibit HPA activity and enhance the neural state of mice through the activation of the ERK pathway [71,72]. Currently, it is understood that BDNF functions by activating the tyrosine protein kinase B receptor (TrkB) on the cell surface, subsequently initiating the ERK signaling pathway. The phosphorylated ERK1/2 molecules can translocate to the nucleus, where they activate the cAMP response element-binding protein (CREB), which in turn regulates the transcription of downstream genes, promotes BDNF production, and exerts an antidepressant effect. Notably, CREB, a nuclear protein that selectively binds to cAMP response elements, stimulates gene transcription and is involved in various neuronal activities, including neuronal cell development, survival, and plasticity. This indicates that CREB has a significant impact on the indirect realization of neural regulation by EGCG. Although it remains unclear whether the antidepressant effect of ERK is primarily due to the upregulation of ERK itself, the activation of the downstream target BDNF, or the phosphorylation of the downstream substrate CREB, it is widely accepted that EGCG exerts its antidepressant effects by modulating the ERK1/2-CREB-BDNF signaling pathway.

EGCG has the potential to modulate the dopaminergic system, thereby producing antidepressant effects. Research indicates that DA is metabolized or inactivated by monoamine oxidase (MAO), located in the mitochondria, and catecholamine ortho-methyltransferase (COMT), found in the cytoplasm. Under the influence of MAO, DA is initially converted into 3,4-dihydroxyphenylacetaldehyde (DOPAL), which is subsequently transformed into 3,4-dihydroxyphenylacetic acid (DOPAC) by aldehyde dehydrogenase. In a 1-methyl-4-phenyl-1, 2, 3, 6-tetrahydropyridine (MPTP)-induced PD mouse model, EGCG (25 mg/kg/day) was shown to inhibit the loss of tyrosine hydroxylase (TH)-positive cells in the substantia nigra (SN) and to prevent the reduction in TH activity in the striatum. Additionally, EGCG preserved levels of DA and its metabolites, DOPAC and homovanillic acid (HVA), in the striatum, effectively safeguarding against monoaminergic disorders within the nervous system [73]. In another PD mouse model, doses of EGCG (25 and 50 mg/kg/day) also mitigated the MPTP-induced (30 mg/kg/day) decline of dopaminergic neurons and inhibited the increase in serum concentrations of TNF-α and IL-6 [74]. These findings suggest that EGCG protects against excessive apoptosis of dopaminergic neurons and reduces pro-inflammatory cytokines in a PD mouse model. Collectively, this evidence highlights the potential antidepressant properties of EGCG through its influence on the monoaminergic system. Other studies have indicated that EGCG can modulate the levels of the dehydroepiandrosterone (DHEA) hormone within the HPA axis, thereby enhancing the body’s immune response while demonstrating no significant effect on CORT levels. Specifically, the concentration of DHEA in the experimental group that received 100 mg/kg of EGCG for 6 weeks exhibited a notable increase (*p* < 0.05); however, CORT levels among the experimental groups (aqueous EGCG at 25, 50 and 100 mg/kg or vehicle control, n = 6 per group) showed no significant differences [75]. EGCG appears to enhance the HPA axis system and regulate the body’s physiological state. Nevertheless, the theoretical mechanisms underlying these effects remain unclear, necessitating further experimental research to elucidate how EGCG influences the regulation of the HPA axis.

(2) EGCG Regulating Nervous System

EGCG primarily achieves a balance in the body’s immune system and exerts an antidepressant effect by regulating cell signal transduction, inhibiting the secretion of inflammatory factors, and suppressing the gene expression of pro-inflammatory proteins. Researchers have compared the abilities of polyphenol compounds from various plants to inhibit neuroinflammatory signals, revealing that the effect of EGCG is particularly significant [137]. The neuroregulatory effect of EGCG largely depends on its capacity to inhibit the activation of microglia, effectively down-regulating the secretion and expression of cytokines as well as the signal transduction functions of its receptors [138]. In cases of nervous system disorders, microglia are typically activated, which may exert either regulatory or toxic effects on the nervous system [139]. Numerous studies have demonstrated that microglia are highly sensitive to external environmental stimuli. In the presence of harmful stimuli, their rapid responses can lead to the upregulation of endogenous inflammatory mediators [140]. Additionally, EGCG can inhibit the activity of iNOS released by microglia. It inhibits the transcription of iNOS and the production of NO by preventing NF-κB from binding to the iNOS promoter, thereby reducing both the activity and protein levels of iNOS in LPS-induced microglia [77]. Consequently, EGCG can indirectly alleviate the damage caused by microglia. Furthermore, the antioxidant effect of the Nrf2-ARE (nuclear factor E2-related factor 2/antioxidant response element) pathway is well recognized. The Nrf2-ARE system plays a crucial role in neuroprotection by regulating the expression of antioxidant molecules and enzymes [141]. EGCG has been shown to induce the expression of Nrf2 protein and reduce the phosphorylation of NF-κB, thereby enhancing the antioxidant response and mitigating neuroinflammation.

Depression has been shown to increase the susceptibility of certain neuronal populations to cell death, and general antidepressant treatments have demonstrated antagonistic effects on this phenomenon [142]. Consequently, a promising approach in the treatment of depression is neuroprotection through the inhibition of apoptosis. Studies indicate that EGCG reduces the expression of Caspase3 and the apoptosis of neurons in the HIP, thereby playing a neuroprotective role [78]. Additionally, EGCG can directly bind to the benzodiazepine binding site of the GABA receptor complex, effectively replacing the endogenous negative regulator of the GABA receptor [79]. It is established that an endogenous negative regulator exists at the benzodiazepine binding site of the GABA receptor in rats [143]; thus, the replacement of these endogenous negative regulators by EGCG may contribute to advancements in antidepressant research. Furthermore, EGCG can help maintain the healthy homeostasis of the nervous system by normalizing the ERK1/2 signaling pathway. Although research on the ERK1/2 signaling pathway is still in its early stages, existing literature suggests that, under the influence of BDNF-induced neural activity, ERK functions more as a target for enhanced neural activity rather than as a mediator of weakening in the context of inflammation. Given the considerable complexity of external signals that promote this pathway, it remains unclear whether EGCG can simultaneously regulate multiple ERK pathways in various directions or what the net effect of EGCG-mediated ERK regulation might be. Nonetheless, it is undeniable that EGCG can exert its antidepressant effects through the ERK signaling node.

(3) EGCG Regulates the Intestinal System

The diverse microbial flora and their metabolic activities in the intestinal tract facilitate bidirectional information transmission through the brain–gut axis, exerting functional effects on both the nervous and immune systems [144]. While changes in gut microbiota are not considered the primary pathogenic factor in depression, they do exert a certain degree of negative impact. Research has demonstrated that EGCG exerts its antidepressant effects by enhancing the intestinal barrier system, regulating the activity of beneficial intestinal bacteria and their metabolites, and altering the composition and structure of the intestinal microbiota.

Studies have shown that EGCG treatment primarily repairs the intestinal barrier by protecting the intestinal mucosal barrier from damage and enhancing the homeostasis of intestinal microbiota, thereby improving inflammatory markers [84]. Upon intake, intestinal microorganisms metabolize EGCG through various degradation pathways, including hydrolysis, C-ring cleavage, and reduction, generating highly bioavailable fission products such as EGC, GA, hydroxyisopropanol, phenyl γ-pentolactone (PVLs), and its related phenylvaleric acids (PVAs) and phenolic acids, which exhibit significant anti-inflammatory, antioxidant, and antibacterial activities. Furthermore, some studies suggest that the decomposition of EGCG by intestinal microbiota may promote the differentiation of nerve cells [85]. The diversity of EGCG metabolites and their inherent biological activities provide a functional basis for their antidepressant effects. Current evidence from various animal models confirms that EGCG treatment can regulate the levels of pro-inflammatory factors (TNF-α, IL-1β, IL-6, MCP-1, MIP-2, IFN-γ) and oxidative stress markers (MPO, NO, ALT, and AST) in the intestine [145]. When an individual consumes tea soup, EGCG is absorbed in the intestines, and it is subsequently distributed to various tissues and organs throughout the body. However, it is noteworthy that the bioavailability of EGCG in the small intestine is only approximately 0.1% [146]. Nonetheless, the bioavailability of oral EGCG can be significantly enhanced through structural modification or the use of nanomaterial protection and delivery systems [134], thus paving the way for potential clinical applications of EGCG.

(4) EGCG Regulates Oxidative Stress

Oxidative stress is believed to result from an imbalance between the production of reactive oxygen species (ROS) in the organism and the antioxidant capacity of cells, leading to a disruption of the equilibrium system that triggers a stress response. Under conditions of oxidative stress, individual brain neurons become susceptible to physiological diseases, including damage and degeneration, neuroinflammation, and mitochondrial dysfunction, due to the excessive presence of ROS. Consequently, ROS is recognized as a critical molecular factor contributing to nerve damage and degenerative diseases [147]. Currently, the system comprising ROS and oxidative stress components is emerging as a significant therapeutic target for mental disorders, such as depression, PD, anxiety disorders, and Alzheimer’s disease, due to its mechanistic effects on brain structure and function, regulation of signaling pathways, neurotransmitter levels, and feedback regulation of various physiological systems, including the HPA axis [148]. Research has demonstrated that EGCG can reverse mitochondrial dysfunction and inhibit oxidative stress through the PGC-1α/SIRT1/AMPK signaling axis [149,150]. The PGC-1 family consists of three co-activators: PGC-1α, PGC-1β, and PGC-1-related coactivator (PRC). Among these, PGC-1α and PGC-1β play essential roles in integrating signaling pathways and maintaining oxidative balance, while PGC-1α specifically is involved in oxidative metabolism, mitochondrial biogenesis, and the regulation of ROS. Numerous studies have indicated a significant decrease in PGC-1α levels in stressed animals. The upregulation of PGC-1α has been shown to mediate improvements in stress management and reductions in depressive behavior, with EGCG and GTP administration significantly alleviating stress states [90,91]. For these reasons, the enhancement of PGC-1α expression in the brains of stressed animals induced by EGCG and GTP may play a crucial role in mediating their effects on neurotransmission, stress, and depression. There exists a regulatory relationship among PGC-1α, AMPK, and SIRT1. AMPK promotes the expression and activation of PGC-1α through a phosphorylation pathway, while SIRT1 deacetylates and activates PGC-1α [151]. Consequently, the PGC-1α/SIRT1/AMPK axis is essential for EGCG to exert its anti-oxidative stress effects and may serve as an effective mechanism for mediating EGCG’s anti-depressive properties.

(5) Tea Polyphenol Mixture Antidepressant

Daily tea drinkers typically consume a mixture of multiple functional active ingredients rather than relying on a single component. In particular, the tea polyphenol mixture elicits specific antidepressant effects by interacting with the gut–brain axis. This axis serves as a crucial two-way communication channel between the immune system and the nervous system, playing a vital role in maintaining the body’s healthy balance. Communication along this axis is facilitated through a complex network of afferent fibers that project to various interconnected regions of the gastrointestinal tract (GI) and the CNS [152]. Within this network, changes in the abundance of gut microbiota can indirectly trigger alterations in brain chemistry, particularly affecting neurotransmitter levels in the nervous system [153]. Recent studies have demonstrated that major short-chain fatty acids (SCFAs) can cross the BBB, alleviate abnormal behaviors induced by psychosocial stress, and exhibit antidepressant and anxiolytic effects in mouse models [154,155]. Furthermore, a human fecal culture experiment revealed that, compared to the control group, catechins EGCG, GCG, and EGCG 3′Me could induce higher concentrations of SCFAs, thereby establishing a unique gut–brain axis system mediated by SCFAs that supports the antidepressant effect of tea polyphenols [156].

Gallic acid, a phenolic acid, acts as a precursor for the synthesis of catechins. Although gallic acid is not the primary functional component in tea, it possesses pharmacological activities, including anti-inflammatory, antioxidant, and antibacterial properties, and shows potential efficacy in treating depression. Gallic acid exhibits neuroprotective properties and can prevent or delay neurological diseases through various mechanisms, thereby promoting human health [157]. Administration of gallic acid reduced the immobility time of rodents in the forced swimming test (FST), achieving its antidepressant effect by inhibiting IAS-induced oxidative stress and reducing acetylcholinesterase (AChE) activity [158]. Additionally, some researchers have suggested that gallic acid may enhance the levels of monoamine substances at synapses, a hypothesis supported by experimental findings: in this study, multiple monoaminergic antagonists counterbalanced the antidepressant effect of gallic acid. Its ability to regulate the monoaminergic system may be linked to its antioxidant activity [159].

#### 2.3.4. Tea Pigments

Due to the structural complexity of TRs and TBs, research on these compounds has been limited, with greater focus currently placed on theaflavins. Studies have indicated that TFs contribute to the recovery of neurological function and the repair of cognitive disorders by reducing oxidative stress, promoting neural stem cell (NSC) proliferation, and preventing apoptosis. These findings suggest that TFs may serve as potential therapeutic agents for neurological disorders [94,95]. Furthermore, TFs exhibit an inhibitory effect on cellular inflammation, primarily by reducing the production of pro-inflammatory cytokines, inhibiting the activation of IκB kinase and NF-κB pathways, and decreasing dendritic atrophy in the PFC and HIP. This action subsequently enhances cognitive ability and alleviates depressive symptoms. Additionally, some studies have reported that the biological efficacy of TFs surpasses that of catechins, chlorogenic acid, or caffeic acid, with their potency being comparable to that of EGCG [96]. However, the physiological mechanisms through which TFs cross the BBB remain unclear. Moreover, TFs may also alleviate depression by modulating the monoaminergic system. Research has demonstrated that the administration of TFs can increase the circulating levels of DA in the frontal cortex of mice, although no significant changes are observed in the HIP. Simultaneously, TF administration does not alter the levels of NE and serotonin in the frontal cortex, suggesting that TFs exert anti-anxiety effects by activating the dopaminergic system [100]. Additionally, reports suggest that the mechanism by which TFs enhance DA neurotransmission may involve mitigating damage caused by free radicals to the DA neurotransmission system and improving the functionality of DA transporters, thereby contributing to their antidepressant effects [].

#### 2.3.5. Tea Saponin

Tea saponin, a principal active component of tea, is classified as an olefin-type pentacyclic triterpene saponin. It serves as a naturally derived inhibitor of NF-κB signaling and has been shown to decrease the incidence of depression. The levels of 5-HT observed in the oral 1% and 5% tea saponin treatment groups over 6 weeks were comparable to those in the positive control group, but significantly lower than those in the model group. Following the administration of tea saponin, the increase in TNF-α and TSLP levels induced by DNCB was mitigated; furthermore, the levels of TNF-α and IL-6 in skin tissue were suppressed. The IL-4 levels in each treatment group showed a statistically significant difference when compared to the DNCB control group, although no significant differences were observed among the treatment groups (*p* > 0.05). Additionally, tea saponins may influence depression through their effects on the gut–brain axis and by alleviating neuroinflammation. In a stress model involving C57BL/6J mice subjected to intragastric administration of a high-fat diet (HFD) for 8 weeks, those treated with oral tea saponin mixed with HFD for 6 weeks exhibited a reduction in alterations to the intestinal microbiota. Moreover, the recognition and memory impairments induced by HFD were effectively prevented, and improvements were noted in neuroinflammation and BDNF deficits in the HIP of mice in the experimental group. This provides compelling evidence for the therapeutic significance of the tea saponin–brain–gut axis [103].

## 3. Discussion

Numerous studies have demonstrated that tea and its bioactive components possess the potential to prevent or treat depression. However, it is important to acknowledge that these studies contain inherent experimental flaws, and the potential biases resulting from these flaws should be carefully considered. To enhance the reliability and validity of future research, several key factors must be addressed. First, relevant epidemiological studies indicate that the preventive effect of tea consumption on depression is limited. This limitation arises because, in addition to tea drinking, other lifestyle factors, such as a healthy diet, physical exercise, and acupuncture, may also contribute to the improvement of depressive symptoms [160,161]. Furthermore, it is crucial to note that a causal relationship cannot be established solely based on cross-sectional studies. Most investigations have employed longitudinal randomized controlled trials to examine the correlation between tea consumption and depression. Existing research suggests that the antidepressant effect of tea components is constrained. In the most favorable scenario, tea can only serve as an adjunctive therapy for the prevention or treatment of depression, which complicates its potential to become a mainstream treatment option. The observed improvement in mood, particularly among healthy individuals, does not provide robust evidence for the clinical efficacy of tea consumption in patients with major depressive disorder. If future studies can confirm a significant correlation between tea and its bioactive components and positive emotional states, or establish tea consumption as an economically effective means of maintaining mental health, this could substantially enhance society’s understanding of the role of tea in the prevention and treatment of depression. Although there may be an inverse relationship between tea consumption and depression [43,45], wherein tea drinking may reduce the incidence of depressive symptoms, the onset of depression may also lead to a decrease in the amount of tea consumed by patients, indicating a complex interaction between the two. Furthermore, contemporary understanding of the causes of depression has evolved beyond the pathogenic factors solely associated with individual physiological dysfunction. External factors, including the social, familial, cultural, and environmental systems surrounding an individual, can also trigger this condition [162]. Consequently, the prevalence of depression complicates treatment efforts even further. Existing research indicates that the primary beneficiaries of tea and its bioactive components are sub-healthy individuals, particularly those exhibiting undiagnosed depression or mild symptoms. However, challenges remain in determining the absolute effectiveness of specific functional components in tea for treating depression. Future research should concentrate on exploring the association network between tea components and depression, with the aim of revealing potential causal relationships or clarifying the mechanisms through which different teas and their components may collaborate to exert antidepressant effects. Additionally, it is important to acknowledge some negative effects associated with tea consumption, such as the overstimulating effects of caffeine, gastrointestinal discomfort, and insomnia resulting from excessive tea intake, while also fully considering the body’s tolerance of the tea drinker.

When analyzing the relationship between tea and depression, researchers must carefully select appropriate experimental materials and representative populations. The content of bioactive compounds in various types of tea varies significantly; for instance, green tea contains approximately 18–42% catechins, whereas the oxidation process during the production of black tea reduces the catechin content to between 3 and 30%, resulting in a greater conversion to tea pigments [163]. This difference naturally influences the antidepressant effects of different types of tea. Moreover, even within the same type of tea, the bioactive ingredients can differ due to variations in the tea tree varieties used, the tenderness of the fresh tea leaves, the planting region, and the processing techniques. Therefore, when researchers seek to clarify the therapeutic effects of a specific type of tea on depression, it is essential to select representative samples of tea as the experimental material. Additionally, the selection of the test group may introduce deviations in the experimental results. The research design must thoroughly consider key factors such as gender, race, and regional differences. Some studies on depression are conducted solely on community or clinical samples, which restricts the generalizability of the findings. Researchers should also consider the age demographics included in these studies, as differences in age groups can lead to variations in physical function, which may potentially skew the experimental results.

When conducting experimental data analysis, selecting an appropriate evaluation method is crucial, as different analytical approaches may lead to deviations in the experimental results. Most drug trials evaluating the efficacy of treatments for depression utilize professional scales, such as the 24-item Hamilton Depression Scale (HAMD-24), the Montgomery–Asberg Depression Rating Scale (MADRS), the Beck Depression Inventory (BDI), and the Social Support Rating Scale (SSRS) [164]. However, while the scores obtained from these scales are statistically significant, they do not necessarily indicate that the actual physiological state of the evaluated individual has improved or that they feel better. In fact, the minimum clinically relevant difference reflected by scale scores remains a controversial topic [165]. Furthermore, scale scores may exhibit subtle changes that appear as significant improvements in statistical analyses but are difficult for clinicians or patients to detect in practice. More effective experimental outcomes should be evidenced by simultaneous improvements in both the clinical examination results of patients’ physiological conditions and the scale scores. This means determining whether the intervention measures have genuinely enhanced the patients’ physical health and the degree of positive mood reported by the patients themselves. Due to inherent differences in diagnostic methods for depression, many current studies rely on self-reported results from participants. Although some studies employ more reliable semi-structured assessment tools or expert clinical diagnoses, this has also resulted in contradictions in data comparisons among existing studies.

Research on the anti-stress effects of tea drinking requires further exploration. Future studies should focus on the interactions among various tea components and the bioavailability of each component. For instance, theanine is sensitive to caffeine and EGCG. The bioavailability of EGCG in the small intestine is only 0.1% [146], while oral GABA faces challenges in crossing the BBB. Researchers must design effective methods to deliver the active ingredients of tea to target locations to optimize efficacy. Additionally, the role of tea-drinking behavior and sociocultural factors warrants examination. Drinking tea is often accompanied by social interactions, where the interplay of verbal communication and behavioral expression elicits corresponding responses in the psychological system. Positive and pleasant emotions can significantly enhance the antidepressant effects of tea. It is essential to recognize that individuals from diverse social and cultural backgrounds have varying interpretations of tea-drinking behavior. Therefore, further research is necessary to clarify the influence of behavioral and sociocultural factors related to tea consumption.

## 4. Conclusions and Perspectives

This review systematically analyzes the potential of bioactive compounds in tea for preventing depression or as an adjunct to existing therapies. Tea and its active ingredients exert an antidepressant effect on consumers through a synergistic mechanism that influences multiple physiological pathways. Among these signaling pathways, the ERK/CREB/BDNF pathway and the GABA-protective pathway are particularly noteworthy. Both pathways are modulated by compounds found in tea and contribute to maintaining homeostasis. Additional mechanisms mediated by tea include the enhancement of SCFA/AMPK signaling, the inhibition of microglial polarization activity, and the promotion of MGBA normalization, with EGCG playing a pivotal role. Furthermore, compounds such as *L*-theanine, tea polyphenols, and tea pigments exert antidepressant effects by normalizing the HPA axis, regulating the monoaminergic system, and increasing BDNF production. These compounds also demonstrate anti-stress effects by boosting the abundance of microorganisms that directly influence the nervous system, such as Lactobacillus and Bifidobacterium. Additionally, tea consumption aids in regulating the immune system, inhibiting the expression of pro-inflammatory factors to alleviate inflammation, and eliminating free radicals while reducing the levels of pro-oxidative factors, thereby mitigating the oxidative stress response. This is a critical component of the antidepressant activity mediated by tea. However, due to the limitations of current research methodologies and experimental data, it remains challenging to ascertain which mechanism of action yields the most significant net effect in the context of depression treatment. This uncertainty may emerge as a key focus for future research. In conclusion, current research findings indicate that moderate tea consumption has a significant preventive or therapeutic effect on depression. We can reasonably predict that, in the future, tea and its derivatives may become viable options for the prevention or treatment of depression.

## Figures and Tables

**Table 1:** The mechanism of tea and its active ingredients for depression.

Tea Ingredients	Theoretical Approach	Action Mechanism	Ref.
*L* -theanine	Improved HPA axis	1. Decreases ACTH, increases cortisol, and inhibits NF-κB phosphorylation	[53]
		2. Inhibits the release of glutamate and decarboxylates glutamate	[54]
	Neuromodulation	1. Increases glycine concentration in striatum	[55]
		2. The concentration of PFC, NAc and AN and the 5-HT, norepinephrine and dopamine in HIP are increased	[56]
		3. Activation of the dopamine D1/5 receptor-PKA pathway improves monoamine transmission in the HIP	[57]
		4. The expression of holo-GR and DNMT3a is inhibited, and the expression of NPAS4 is indirectly promoted	[58]
		5. Indirectly reduces the intermittent arousal and improves sleep quality	[59]
		6. Inhibits the formation of AGE and regulate Sirtuin1 and BDNF signaling pathways	[60]
	Immunomodulation	1. Reduces inflammation in many types of cells	[61,62]
		2. Inhibit of NF-κB pathway to reduce inflammatory cytokine levels	[63]
		3. Ameliorate intestinal pathological injury and inhibit various inflammatory cytokines	[64]
GABA	Improved HPA axis	1. GABA inhibits glutamate release and reduces the activation of CRF neurons	[65]
		2. GABA promotes recurrent GABAergic synaptic connections between PVN CRFR1 and CRF neurons	[66]
	Neuromodulation	1. GABA system activation promotes neuronal signal transmission	[67]
	Intestinal regulation	1. Long-term administration of Lactobacillus rhamnosus JB-1 improves GABA receptor expression in the cortico-hippocampus, thereby reducing CORT and depressive behaviors	[68]
Flavonoids		1. Protects neurons from oxidative stress, inhibits neuroinflammation and improves signaling pathways	[69]
EGCG	Improved HPA axis	1. Reduces CORT, CRH, and ACTH levels	[70]
		2. Activation of ERK pathway inhibits HPA activity and improves neural status in mice	[71,72]
		3. Protects the level of DA and its metabolites and prevents monoaminergic disorders in the nervous system	[73]
		4. Prevents the decrease in dopaminergic neurons and inhibits the increase in serum concentrations of TNF-α and IL-6	[74]
		5. Regulates DHEA hormone levels in the HPA axis	[75]
	Neuromodulation	1. Inhibits the phosphorylation of NF-κB and STAT3 in the hypothalamus and inhibits the production and release of TNF-α, IL-6 and IL-1β	[76]
		2. The binding of NF-kB to iNOS promoter is prevented, thus inhibiting the transcription of iNOS to produce NO and reducing the activity and protein level of iNOS in microglia	[77]
		3. Decreases the expression of Caspase 3 in HIP and apoptotic neurons	[78]
		4. Increasing the ratio of NGF/proNGF can improve the relative expression level of NGF, inhibit Aβ deposition and neuronal apoptosis in HIP	[78]
		5. The full function of GABA receptors may be restored by substituting DBI type antianxiety ligands	[79]
	Immunomodulation	1. Neuroimmunological coordination is achieved through NF-κB, PI3k-Akt-mTOR, and NO pathways	[80]
		2. Decreases levels of IL-1β and TNF-α in the HIP	[70]
		3. The levels of TNF-α, IL-1β, IL-6 and IL-8 are inhibited	[81]
		4. Inhibition of NFκB/ activator protein 1 (AP-1) pathway shows anti-inflammatory effects	[82]
		5. Inhibits the activation of ERK1/2, P38MAPK, and NF-κB and reduces the production of inflammatory chemokine IL-8 in the supernatant of AC16 cardiomyocytes	[83]
	Protect intestinal health	1. Protects intestinal mucosal barrier from damage and improves intestinal microbial homeostasis	[84]
		2. Decreases plasma lfructose–rhamnose–ratio and plasma sucralose-to-erythrolitol ratio, decreases colon protein levels of IL-1β, IL-6, and TNF-β, and colon lipid peroxides.	[85]
		3. Up-regulates SCFA level in cecal contents of mice	[84]
		4. The levels of cortisol, ACTH, and CRF are significantly decreased, and the production of intestinal SCFAs is induced	[86]
		5. Enriches SCFAs producing bacteria	[87]
		6. TotM connects the gut microbiota formed after ingesting EGCG with host neurons and motor function	[88]
		7. Enhances the protective effect of intestinal structure and decreases the level of 5-HT in the colon; increases the level of 5-HT in the HIP or inhibits the apoptosis of hippocampal neurons	[89]
	Reduce oxidative stress	1. Combined administration of EGCG and green tea polyphenols can up-regulate PGC-1α	[90,91]
		2. EGCG activates PGC-1α by promoting AMPK phosphorylation	[92,93]
		3. EGCG can regulate oxidative stress and neuroimmunity through the NO pathway, can regulate the expression levels of genes including iNOS, COX-2 and TNF-α, and can prevent the binding of NF-kB to iNOS promoters	[77,80]
Tea pigments		1. TFs increases overall antioxidant capacity and decreases OS levels	[94]
		2. Theaflavin administration significantly increases Nrf2 protein and Nrf2 mRNA levels, and TF treatment can reduce the apoptosis rate of CI/RI rats	[95]
		3. Inhibiting the production of inflammatory cytokines significantly reduces depressive behavior in mice	[96]
		4. Inhibition of NF-κB activation significantly reduce IL-1β, TNF-α, and IL-6 secretion in a dose-dependent manner	[97]
		5. TFDG inhibits the expression of TNF-α, IL-1β and IL-6	[98,99]
		6. The circulating level of DA in the frontal cortex of mice is increased	[100]
Tea saponin		1. BALB/c mice can significantly improve the symptoms of skin inflammation and significantly reduce the level of inflammatory factors in serum and skin tissue	[101]
		2. The levels of inflammatory signaling molecules, proinflammatory cytokines, and inflammatory signaling molecules in adipose tissue and liver of obese mice are decreased	[102]
		3. The changes in the intestinal microbiota of mice are reduced, the recognition and memory disorders are effectively prevented, and the neuroinflammation and BDNF defects in the HIP of the experimental group of mice are improved	[103]

Abbreviations: ACTH, adrenocorticotropin; BDNF, brain-derived neurotrophic factor; CRF, corticotropin-releasing factor; DHEA, dehydroepiandrosterone; DNMT3a, DNA methyltransferase 3A; EGCG, (-)-epigallocatechin-3-gallate; GABA, γ-aminobutyric acid; HIP, hippocampus; 5-HT, 5-hydroxytryptamine; IL, interleukin; NAc, nucleus accumbens; NPAS4, neuron PAS domain protein 4; PFC, prefrontal cortex; SCFAs, short-chain fatty acids.

## Data Availability

No new data were created or analyzed in this study. Data sharing is not applicable to this article.
